# Quantum decision making in automatic driving

**DOI:** 10.1038/s41598-022-14737-2

**Published:** 2022-06-30

**Authors:** Qingyuan Song, Weiping Fu, Wen Wang, Yuan Sun, Denggui Wang, Jincao Zhou

**Affiliations:** 1 Xi’an University of Technology, Xi’an, China; 2 Xi’an International University, Xi’an, China; 3 Huanghe Science and Technology College, Zhengzhou, China

**Keywords:** Engineering, Physics

## Abstract

The behavior intention estimation and interaction between Autonomous Vehicles (AV) and human traffic participants are the key problems in Automatic Driving System (ADS). When the classical decision theory studies implicitly assume that the behavior of human traffic participants is completely rational. However, according to the booming quantum decision theory in recent years and actual traffic cases, traffic behaviors and other human behaviors are often irrational and violate the assumptions of classical cognitive and decision theory. This paper explores the decision-making problem in the two-car game scene based on quantum decision theory and compares it with the current mainstream method of studying irrational behavior-Cumulative Prospect Theory (CPT) model. The comparative analysis proved that the Quantum Game Theory (QGT) model can explain the separation effect which the classical probability model can’t reveal, and it has more advantages than CPT model in dealing with game scene decision-making. When two cars interact with each other, the QGT model can consider the interests of both sides from the perspective of the other car. Compared with the classical probability model and CPT model, the QGT is more realistic in the behavior decision-making of ADS.

## Introduction

For a long time in the future, autonomous vehicles will inevitably share urban roads with human traffic participants^1^. In order to drive safely and efficiently in this complex traffic surrounding, autonomous driving vehicles need to correctly estimate the behavioral intention of human traffic participants and interact with human traffic participants naturally just like human driving vehicles^2,3^. The behavior of human traffic participants and their interactions are very random in the real world actually. Osamu proposed that such randomness is characterized by obvious uncertainty and irrationality^4^. The "long tail" problem of autonomous driving includes various fragmented scenarios, extreme situations and unpredictable human behavior. This is related to the unreasonable behavior intention and uncertainty^5^, which needs to be studied by correct and effective cognitive and decision theory.

The mainstream methods of behavior decision-making, the traditional machine learning methods based on classical probabilistic reasoning^6,7^ and the deep learning methods based on data drive are common. Traditional machine learning methods generally assume that the evolution process of traffic participants has the characteristics of Markov Decision Processes (MDP), Hidden Markov Model (HMM), Dynamic Bayesian network (DBN) and other methods to infer intentions. And the most extensive Partially Observable Markov Decision Processes (POMDP) is used to obtain correct behavior decision. However, the existing results of human behavior decision-making theory show that human behavior is incompatible with the complete rational hypothesis in classical decision-making theory^8^. And the cognitive and decision-making theory based on classical probability cannot accurately describe human behavior and its interaction^9^, which are the main bottlenecks restricting the safe and efficient driving of autonomous driving in actual urban traffic scenes^10^. Data-driven deep learning methods must rely on massive big data sample training (accumulated actual driving miles need to reach tens of billions of miles) to deal with the “long tail” problem that may cause driving accidents^11,12^ and the explosive progress of autonomous driving technology being hindered. Daily, low probability of occurrence or emergent, dangerous, and the edge (corner) scene of scarce samples are often related to irrational behavior and interaction^13,14^. And pure deep learning method based on data driven is difficult to effectively cope with the “long tail” problem^15^.

What is gratifying is that quantum theory originated in the field of microscopic physics has been extended in the past two decades and it made great progress in many non-physical and macro fields such as cognition, decision making, information, communication, computing, etc. It has not only formed an increasingly mature theoretical system, but also been increasingly widely applied^16^. In particular, the initial quantum inkling in the field of mobile robots, being most closely related to unmanned driving technology^16^, allows us to see the potential and possibility of applying quantum theory to solve the cognitive problems of autonomous driving. Quantum theory provides a new way to study the uncertain behavior (including irrational behavior) of human traffic participants and their interaction. How to correctly understand the uncertain behavior and interaction of human traffic participants based on quantum theory, and how to make correct interactive behavior decisions based on this is the focus of this paper waiting to explore and solve.

At present, there is no relevant literature to explain autonomous driving with quantum cognition and decision theory. Based on the Quantum Game Theory (QGT) model, this paper analyzes the behavior decision-making problem of human traffic participants (two-car game case), and compares it with the current mainstream method of irrational behavior research-Cumulative Prospect Theory (CPT), verifying the accuracy of the two models with data set experiments.

## Related work

### Decision-making of automatic driving behavior considering interaction

The most widely used traditional machine learning is Partially Observable Markov decision processes (POMDP)^17^, including prediction intention and behavior selection, which regards the intention of road users as a potential representation in decision-making state space. Mehta^18^ proposed a navigation method based on POMDP, the evaluation decision was made by a set of pre-designed forward simulation predictions of closed-loop behaviors, and the social interaction between autonomous vehicles and other participants was also considered. Hubmann^19^ proposed a unified decision-making planning framework of online POMDP for complex signalless intersections, which considered the uncertainty of traffic participants' behavior intention, motion prediction and interaction with autonomous vehicles, but like most literatures, it still assumed that traffic participants' behavior decisions were rational. There are also some behavioral decision-making frameworks based on game theory that consider interaction, but the behavioral intention cannot be considered, or the participants are assumed to be rational. Fabiani^20^ proposed a multi-vehicle distributed hybrid coordination decision-making framework, which formalized the problem of non-cooperative behavior coordination as a generalized mixed integer game model. Coskun^21^ combines game theory with MDP, and describes the decision-making process of interaction between autonomous vehicles and other vehicles with MDP game model. Isele^22^ formalizes the interactive decision-making process as a random game (extended MDP) model, and realizes interactive decision-making by designing effective search and reasoning strategies of intent game tree. Tian^23^ established an interactive decision-making framework based on K-level game theory, while Li^24^ proposed a game theory framework integrating cognitive hierarchy theory and Bayesian reasoning, which can better describe the bounded rational interaction of participants. In addition, there is a negotiation framework based on heuristic strategy, which is different from the game decision-making framework, and can deal with interactive behavior decision-making problems^3^, but does not consider the irrational behavior of traffic participants.

In recent years, the method of deep learning motion planning based on data-driven has attracted more and more attention^25^. The two most representative paradigms of deep learning in motion planning are planning based on Deep Imitation Learning (DIL) and Deep Reinforcement Learning (DRL)^26^. DIL can be trained with data collected from the real world, but these data are scarce in corner cases, which makes the response of the trained network uncertain when encountering invisible data; DRL system can explore different driving situations in the simulated world, but when transplanted to the real world, these models often have biased behaviors^12^. In recent years, DRL and DIL have provided effective solutions to solve MDP problems in high-dimensional state space and decision space, and promoted the research on autonomous driving behavior decision-making to make important progress^27^. However, the established DRL/DIL model is difficult to verify and ensure safety in the actual complex environment. In the actual deployment of this model, there may be great challenges in terms of stability and robustness^26^, at present, it is basically in the stage of simulation research, and most of them are aimed at simple scenes^12,27,28^. Transferring the driving strategies learned from simulation to the real world is still an open research challenge^29^.

At present, people have made quite a lot of research achievements in the behavioral decision-making planning of autonomous driving and safe navigation, but most of the researches are based on rational behavior assumptions, especially the interaction between autonomous vehicles and traffic participants^24,30^, and their uncertain behavior decisions are mostly based on the classical probability decision-making theory and game theory, but the complete rationality assumed by the classical game theory cannot accurately describe human decision-making behavior^31^.

### Quantum decision theory

Quantum mechanics is the greatest discovery in the last century, which greatly promotes the development of modern science and technology and becomes the theoretical pillar of emerging science and technology. Scholars in the field of cognition have found that the interaction between interference and entanglement in quantum mechanics and human cognition has many similar characteristics, which urges us to construct a mathematical expression method of quantum mechanics, introduce quantum probability into the cognitive field, try to use the unique characteristics of quantum mechanics to build a cognitive model, and explain the problems in the field of human cognition that can not be explained by the cognitive decision theory based on classical probability,quantum cognitive decision theory based on quantum probability is gradually born^32^. Quantum logic was put forward by the famous mathematician Von Neumann, who defined the event as a subspace in Hilbert space^32^, so that quantum probability does not need to be constrained by many Boolean logic rules such as the rule of total probability. Therefore, quantum decision theory can allow those events that violate the law of total probability to exist. Busemeyer and Bruza pointed out that quantum logic is actually a kind of generalized Boolean logic, which does not have many constraints in Boolean logic, has greater flexibility and randomness, and is more conducive to explaining people's judgments and decisions^16^.

In recent 10 years, quantum cognitive decision theory has made a series of breakthroughs in the field of human cognition, which has been recognized as a new way to explore human cognitive science^33–36^. The quantum cognitive decision theory [such as Quantum Bayesian Theory (QBT)^16^, Quantum Game Theory(QGT)^16^, etc.] produced by combining quantum probability with classical machine learning theories (MDP, POMDP, DBN, HMM, etc.) provides a more advanced, effective and feasible theoretical tool for the cognitive decision research of autonomous driving system. Song and others for the first time quantum cognitive decision making theory is introduced into Autonomous driving field, aimed at the pedestrian crossing in Autonomous driving scene to build the kind of QLBN model, and compared with classical bayesian model made, in the case of the pedestrian crossing well explain the existence of irrational behavior, at the same time to build the kind of Quantum social force model and compared with the mainstream data-driven model, it also has a good advantage in pedestrian crossing trajectory prediction^37^, Catarina used QLBN to analyze "prisoner's dilemma" cases and obtain predictive results for similar events. Comparing with reality, Catarina proved the predictability of the quantum probability method^38^.

To sum up, there is no systematic method for automatic driving decision-making considering irrational behaviors of human traffic participants and their interactions. Although quantum decision-making theory has made great progress in recent years, it provides a new method to study the automatic driving decision-making problem considering the interaction of human traffic participants' behaviors (including irrational behaviors), but there are no research cases applied in the field of automatic driving at present. In this paper, the two-car game case is analyzed by QGT, which is the first attempt to apply quantum decision theory in the field of ADS.

## Method

### Classical probability and quantum probability

Let's assume that a system has attribute A, and its value can be up and down. In addition, the system also has attribute B, and its value can be left and right. The biggest difference between quantum probability and classical probability is that there are incompatible attribute pairs, that is, two attributes cannot be measured at the same time. Correspondingly, if two attributes can be measured at the same time, they constitute a compatible attribute pair. For the measurement of an attribute, quantum probability and classical probability will get exactly the same result. Furthermore, for compatible attribute pairs, there is still no difference between quantum probability and classical probability. In other words, the compatible attribute operation in quantum probability has been able to cover all the contents of classical probability theory. However, for incompatible attribute pairs, many classical probability algorithms are no longer valid. The properties of classical probability system can be found in the measurement of compatible attributes of quantum probability, but conversely, the incompatible attributes in quantum probability have special properties, so it can be said that quantum probability contains more probability operation systems than classical probability.

The advantages of quantum probability methods in decision-making will be demonstrated below by comparing Bayesian Network (BN) and Quantum-like Bayesian Network (QLBN) .

#### Bayesian network (BN)

A Bayesian Network (BN) is a directed acyclic graph in which each node represents a random variable, and each edge represents a direct influence from the source node to the target node. The graph represents independence relationships between variables, and each node is associated with a conditional probability table that specifies a distribution over the values of a node given each possible joint assignment of values of its parents.

Bayesian networks can represent essentially any full joint probability distribution, which can be computed using the chain rule for Bayesian networks. Let *G* be a BN graph over the variables ${X}_{1},{X}_{2},...{X}_{N}$. We say that a probability distribution, *Pr*, over the same space factorises according to *G*, if *Pr* can be expressed as the product^39^.

1 $${P}_{r}({X}_{1},{X}_{2},...{X}_{N})=\prod_{i=1}^{N}{P}_{r}({X}_{i}\left|{Pa}_{{X}_{i}}\right.)$$

In Eq. (1), ${Pa}_{{X}_{i}}$, corresponds to the all the parent variables of ${X}_{i}$. The graph structure of the network, together with the associated factorisation of the joint distribution allows the probability distribution to be used effectively for inference (i.e. answering queries using the distribution as our model of the world). For some query Y and some observed variable e, the exact inference in Bayesian networks is given by

2 $${P}_{r}(Y\left|E=e\right.)=\alpha {P}_{r}(Y,e)=\alpha \sum_{\omega \in W}{P}_{r}(Y,e,\omega ), \;\;\;where \;\;\; \alpha =\frac{1}{\sum_{y\in Y}{P}_{r}(Y,e)}$$

Each instantiation of the expression ${P}_{r}(Y=y,e)$ can be computed by summing out all entries in the joint that correspond to assignments consistent with y and the evidence variable e. The random variable W corresponds to variables are neither query nor evidence. The α parameter corresponds to the normalisation factor for the distribution ${P}_{r}(Y,e)$. This normalisation factor comes from some assumptions that are made in Bayes rule^40^.

#### Quantum-like Bayesian network (QLBN)

QLBN can be defined by a pair $\langle G,{P}_{g}\rangle$ where $G$ is a directed acyclic graph represented by a pair $G=(V,E)$. Each vertex ${v}_{i}\in V$ represents a random variable, and the random variable is a quantum state in the complex Hilbert space *H*_*G*_, but ${e}_{j}\in E$ is a set of directed edges that represent the relationship between vertices. ${P}_{g}$ is a density operator in the compound state of independent complex Hilbert Spaces with different dimensions, and ${P}_{g}$ is defined on a fixed basis, so it satisfies the same conditional independence constraint as BN, except that the actual probability value is replaced by the complex probability amplitude^40^.

Quantum probabilities are computed using projective rules that involve three steps. First, the probabilities for all events are determined from a state vector $\left|z\right.\rangle$ ∈ H of unit length (i.e.,${\left|\left|z\right.\rangle \right|}^{2}$ = 1). This state vector depends on the preparation and context (person, stimulus, experimental condition). More is said about this state vector later, but for the time being, assume it is known. Second, to each event there is a corresponding projection operator ${P}_{x}$ that projects each state vector $\left|z\right.\rangle$ ∈ H onto event. Finally, probability of an event is equal to the squared length of this projection:

3 $${Pr}(X)={\left|{P}_{x}\left|z\right.\rangle \right|}^{2}={({P}_{x}\left|z\right.\rangle )}^{\dagger}({P}_{x}\left|z\right.\rangle )=\langle z|{{P}_{x}}^{\dagger}\left.{P}_{x}\right|z\rangle=\langle z|{P}_{x}\left.{P}_{x}\right|z\rangle=\langle z|\left.{P}_{x}\right|z\rangle$$

**Figure 1:**
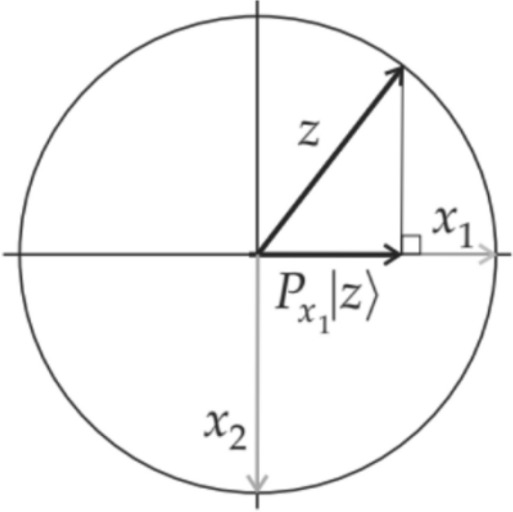
Projective probability: $$\mathit{Pr}({x}_{1})={\left|{P}_{{x}_{1}}\left|z\right.\rangle \right|}^{2}$$.

Projection operators are characterized as being Hermitian and idempotent. To say P is Hermitian means that $P={P}^{\dagger}$; in matrix terms, for every *i* and *j*, the entry ${P}_{i,j}$ in row *i*, column *j* of $P$ and the entry ${P}_{j,i}$ in row *j*, column *i* of $P$ are complex conjugates of each other. To say $P$ is idempotent means ${P}^{2}=P$. Figure 1 illustrates the idea of projective probability. In Fig. 1, the squared length of the projection of $\left|z\right.\rangle$ onto the event is the probability of the event given the state $\left|z\right.\rangle$.

In^40^, Jerome R. Busemeyer carried out a detailed derivation of the quantum probability distribution of single variable and multiple variables, which will not be repeated in this paper. QLBN is described in more detail below.

Let ${H}_{G}$ be a complex Hilbert space representing a QLBN, and let ${H}_{1}\in {H}_{G}, {H}_{2}\in {H}_{G},\dots ,{H}_{n}\in {H}_{G}$ be a collection of different Hilbert Spaces that make up QLBN. The network ${H}_{G}$ of these Hilbert Spaces is defined as the tensor product of each Hilbert space: ${H}_{G}={H}_{1}\otimes{H}_{2}\otimes \cdots \otimes{H}_{n}$. The dimension of ${H}_{G}$ corresponds to the size of the full joint probability distribution. The random variables that constitute the network are represented by quantum states. This means that the random variables are represented as complex probability amplitudes rather than real numbers in BN. In QLBN, two types of quantum states need to be distinguished: (1) the state corresponding to the root node and (2) the state corresponding to the child node. The root node corresponds to the quantum pure state. It can be described by the following formula:

4 $$|{\psi }_{X1}\rangle ={\alpha }_{0}|0\rangle +{\alpha }_{1}|1\rangle ,\mathrm{ Where},{\left|{\alpha }_{0}\right|}^{2}+{\left|{\alpha }_{1}\right|}^{2}=1$$

**Figure 2:**
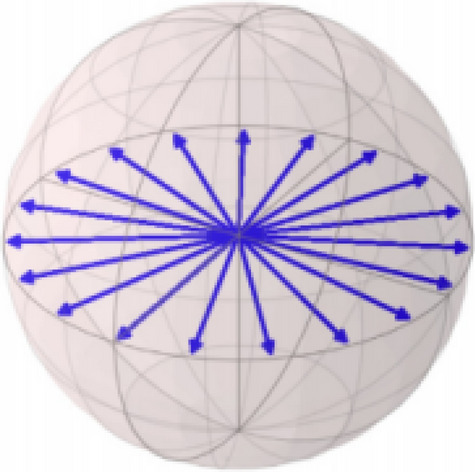
Representation of the pure state (root node).

**Figure 3:**
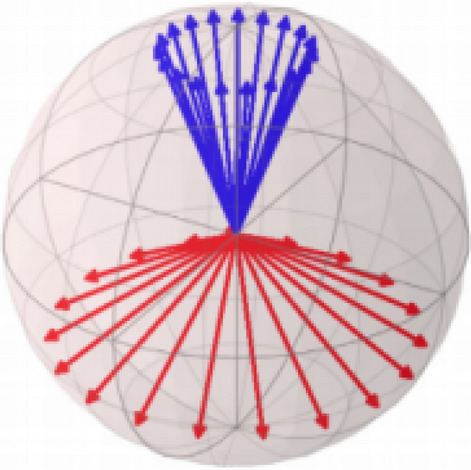
Representation of the state set (child nodes).

And the $|0\rangle$ and $|1\rangle$ is called the basis state and corresponds to the basis: ${\left[\mathrm{1,0}\right]}^{T}$ and ${\left[\mathrm{0,1}\right]}^{T}$. The variable ${\alpha }_{0}$ and ${\alpha }_{1}$ corresponds to the complex probability amplitude of the form: $\sqrt{r}{e}^{i\theta }$, $r\in \mathcal{R}$. On the other hand, the child nodes represent the statistical distribution of different quantum states. This indicates that the child node is represented as a set, in which the conditional probabilities are different quantum states in the set^41^. It can be described in Figs. 2 and 3. Figure 2 shows the representation of the pure state (root node), and Fig. 3 shows the representation of the set of states (child nodes). Among them, since quantum probability amplitudes are represented in complex numbers, a quantum state can be represented geometrically in different ways depending on the phase of the complex amplitudes $\theta$.

In Quantum theory, all independent quantum states contained in a Hilbert space are defined by a superposition state, represented by the quantum state vector $|S\rangle$, which contains the occurrence of all the events of the system. This can be analogous to the full joint probability distribution of classical probability, except that the probability is expressed by the complex probability amplitude instead of the real numbers. In this sense, superposition state $|{S}_{g}\rangle$ contains all possible events in Hilbert space ${H}_{G}$, given by:

5 $$|{S}_{g}\rangle =\sum_{{k}_{1},{k}_{2},...,{k}_{n}}\prod_{j\in G}{\Psi }_{x=j\left|{P\mathcal{a}}_{{\psi }_{x=j}}\right.}|{k}_{1}\rangle \otimes |{k}_{2}\rangle \otimes \ldots \otimes|{k}_{n}\rangle .$$

where ${k}_{1},{k}_{2},\ldots,{k}_{n}$ corresponds to the basis of each quantum state in the network.

The purpose of the density operator ${P}_{g}$ is to describe a system in which we can calculate the probability of finding each state in the network. The implementation method is to calculate the density operator through the cross product of superposition states $|{S}_{g}\rangle$^41^:

6 $$\begin{aligned} {P}_{g}&=|{S}_{g}\rangle \langle {S}_{g}|=\left(\begin{array}{ccc}\begin{array}{cc}{\left|{\alpha }_{0}\right|}^{2}& {\alpha }_{0}{{\alpha }_{1}}^{*}\\ {\alpha }_{1}{{\alpha }_{0}}^{*}& {\left|{\alpha }_{1}\right|}^{2}\end{array}& \cdots & \begin{array}{c}{\alpha }_{0}{{\alpha }_{n-1}}^{*}\\ {\alpha }_{1}{{\alpha }_{n-1}}^{*}\end{array}\\ \vdots & \ddots & \vdots \\ \begin{array}{cc}{\alpha }_{n-1}{{\alpha }_{0}}^{*}& {\alpha }_{n-1}{{\alpha }_{1}}^{*}\end{array}& \cdots & {\left|{\alpha }_{n-1}\right|}^{2}\end{array}\right), \\ & \quad \quad \mathrm{Where } \;\; {\left|{\alpha }_{0}\right|}^{2}+{\left|{\alpha }_{1}\right|}^{2}+\cdots+{\left|{\alpha }_{n-1}\right|}^{2}=1\end{aligned}$$

The density operator ${P}_{g}$ corresponds to a $n\times n$ Hermitian matrix, where *n* is the number of quantum states in the network, which contains the full joint probability distribution of classical probability if we sum the elements of the main diagonal.

7 $$Diag({P}_{g})=Pr({X}_{1},...,{X}_{n})=\prod_{i=1}^{n}Pr({X}_{i}\left|{Pa}_{{X}_{i}}\right.)$$

**Figure 4:**
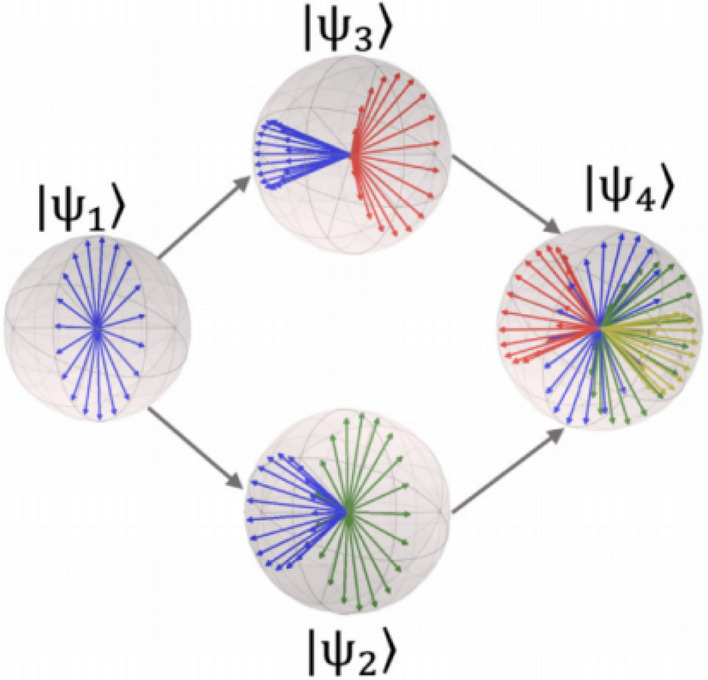
QLBN use quantum states instead of nodes.

Density operators also contain quantum interference terms in non-diagonal elements, which are at the heart of the model. It is through these non-diagonal elements that one can obtain the quantum interference effect during inference, thus deviating from completely rational probabilistic reasoning. It can be seen that quantum states in QLBN allow different levels of deterministic representation, which can be concreted in Fig. 4: $|{\psi }_{1}\rangle$ is a perfectly rational and optimal decision (completely classic), to follow the expected utility axiom (closely related to the rational choice theory in economics)^42^, $|{\psi }_{2}\rangle$ and $|{\psi }_{3}\rangle$ for the prediction of sub-optimal decisions deviating from the expected utility theory, but still provide the satisfaction of the utility (associated with bounded rationality theory), $|{\psi }_{4}\rangle$ for irrational decision^43^ (Quantum) completely, reflects decision choices that lead to less efficient use (associated with contradictory decisions and cognitive biases).

In the process of inference, subgroups of the quantum system need to be traced from the large system represented by the density operator ${P}_{g}$, and partial tracking algorithm is used according to^41^:

8 $${P}_{g}(X)={Tr}_{Y}[{P}_{g}(X,Y)]$$

At the same time, the calculated complex probability amplitude is converted to the actual probability value. Given a certain evidence variable *e*, the quantum edge probability of the discrete random variable is obtained, and the scores obtained are normalized:

9 $$\mathit{Pr}(X|e)=\alpha {\left|\sum_{y}\prod _{k=1}^{N}\psi ({X}_{k}|Parents({X}_{k}),e,y)\right|}^{2}$$

According to the expansion of the above formula, the quantum marginalization formula (10) is obtained, which is composed of two parts: the first part represents the classical probability, and the second part represents the quantum interference term, which is expressed by Formula (11):

10 $$\mathit{Pr}(X|e)=\alpha {\sum _{i=1}^{\left|Y\right|}\left|\prod _{k}^{N}\psi ({X}_{k}|Parents({X}_{k}),e,y=i)\right|}^{2}+2\cdot Interference$$

11 $$\begin{gathered} Interferencr = \hfill \\ \sum\limits_{i = 1}^{{\left| {Y - 1} \right|}} {\sum\limits_{j = i + 1}^{\left| Y \right|} {\left| {\prod\limits_{k}^{N} {\psi (X_{k} |Parents(X_{k} ),e,y = i)} } \right|} } \cdot \left| {\prod\limits_{k}^{N} {\psi (X_{k} |Parents(X_{k} ),e,y = j)} } \right| \cdot \cos (\theta_{i} - \theta_{j} ) \hfill \\ \end{gathered}$$

In the above formula, if ${\theta }_{i}-{\theta }_{j}=\pi /2$, then $cos({\theta }_{i}-{\theta }_{j})=0$, it means that the quantum interference term is canceled and the QLBN collapses into a classical BN. In other words, we can think of QLBN as a more general and abstract model of classical networks because it represents both classical and quantum behavior.

For normalization purposes, we assume that the decision maker is subjected to the same quantum interference term, i.e. $({\theta }_{i}-{\theta }_{j})=\theta$. If $cos\theta =1$, then $\theta =0+2k\pi ,k\in Z$, this is equivalent to the maximum phase-long interference that can be achieved by quantum probabilistic inference. Similarly, at that time $cos\theta =-1$, $\theta =\pi +2k\pi ,k\in Z$, the minimal destructive interference is achieved, at that time $\theta \in [0,\pi ]$, the probability inference calculated by using quantum probability theory can have different ranges of all possible probability values. Therefore, the size of the value $\theta$ represents the uncertainty in the decision-making process.

To sum up, quantum probability has wider physical meaning and properties than classical probability. Measurement is an important way to transform the illusory world of quantum into the real world, and human consciousness itself is transforming various possibilities into reality. This makes many scientists and philosophers think that quantum probability can not only describe the microscopic particle world, but also describe human consciousness and cognitive behavior^39^.

### Comparison of the three models (classical game model, CPT model and QGT model)

In the context of related work in Part II, quantum decision model is the method of this paper to try to solve the irrational behavior and interaction of human traffic participants in autonomous driving. The following paper will compare the three models, so as to further illustrate the necessity of adopting quantum decision method in this paper.

#### Game model based on Markov (classical game model)

Assuming that two decision makers T and I play a game, the strategies that can be adopted are **p** and **y**. The income matrix is constructed as follows (Table 1):

**Table 1:** The payoff matrix of both sides in the game.

	Decision maker T adopts strategy **p**	Decision maker T adopts strategy **y**
Decision maker I adopts strategy **p**	I: 10, T: 10	I: 25, T: 5
Decision maker I adopts strategy **y**	I: 5, T: 25	I: 20, T: 20

All the situations faced by decision makers are defined in a space, which includes four ground States that decision makers may encounter^16^: I_p_T_p_, I_p_T_y_, I_y_T_p_ and I_y_T_y_, where I_i_T_j_ is that decision maker T made the decision of action j after decision maker I taking action i, subscript ‘p’ indicates taking strategy **p**, and ‘y’ indicates taking strategy **y**. These four ground States constitute a four-dimensional dynamic Markov model, which is independent of each other and represents all possible beliefs and behaviors of decision makers. Mathematically, these four ground states are regarded as four basis vectors of a Hilbert space, which are represented by a column vector:

12 $$\psi =\left[\begin{array}{c}\begin{array}{c}\begin{array}{c}{\psi }_{pp}\\ {\psi }_{py}\end{array}\\ {\psi }_{yp}\end{array}\\ {\psi }_{yy}\end{array}\right]$$

This column dimension shows the probability of all possible situations, ${\psi }_{ij}$ shows the probability of decider T in I_i_T_j_ and ${\sum }_{i}{\sum }_{j}{\psi }_{ij}=1$. The following assumptions are made: when decision maker T doesn't know I's behavior, the probability of each ground state is the same, that is, 0.25. When decision maker T knows I's behavior, the corresponding ground States are equally distributed. Then the initial behavior state vector of decision maker T is:

13 $${\psi }_{0}\left(0\right)=\left[\begin{array}{c}\begin{array}{c}\begin{array}{c}0.25\\ 0.25\end{array}\\ 0.25\end{array}\\ 0.25\end{array}\right], {\psi }_{1}\left(0\right)=\left[\begin{array}{c}\begin{array}{c}\begin{array}{c}0.5\\ 0.5\end{array}\\ 0\end{array}\\ 0\end{array}\right],{\psi }_{2}\left(0\right)=\left[\begin{array}{c}\begin{array}{c}\begin{array}{c}0\\ 0\end{array}\\ 0.5\end{array}\\ 0.5\end{array}\right]$$

where, ${\psi }_{0}\left(0\right)$, ${\psi }_{1}\left(0\right)$, ${\psi }_{2}\left(0\right)$, respectively, represents the initial behavior state vector of decision maker T who does not know I's behavior, decision maker T who knows I's intention to adopt strategy **p** and decision maker T who knows I's intention to adopt strategy **y**, after time t, the initial vector will become the final vector ${\psi }_{0}\left(t\right)$, ${\psi }_{1}\left(t\right)$, ${\psi }_{2}\left(t\right)$, it also represents the completion of the decision. This dynamic process can be described by the solution of Kolmogorov forward equation:

14 $$\psi \left(t\right)={e}^{t{K}_{A}}\psi \left(0\right)$$

where, K_A_ is the strength matrix, which is the key to the solution of the equation and is related to the income matrix under different conditions. Finally, the strength matrix is described as:

15 $${K}_{A}=\left[\begin{array}{cc}{K}_{Ap}& 0\\ 0& {K}_{Ay}\end{array}\right],{K}_{Ai}=\left[\begin{array}{cc}-1& {u}_{i}\\ 1& {-u}_{i}\end{array}\right]$$

where, ${u}_{i}$ are utility functions related to the difference between the benefits of decision makers under different decision-making conditions. In classical Markov dynamic decision-making, the value is limited to positive real numbers^44^. For example, the meaning of ${u}_{p}$ is expressed as equation:

16 $${u}_{p}=u({X}_{pp}-{X}_{py})$$

where, ${X}_{pp}$ and ${X}_{py}$ respectively represent the profit value of decision maker T adopting strategy **p** and **y**, after decision maker T knows that I adopts strategy **p**, in Table 2, ${X}_{pp}$ = 10 and ${X}_{py}$ = 5, so ${u}_{p}$ = ${u}_{y}$ = $u(5)$.

**Table 2:** Comparison of the success rates in CPT model and QGT model.

Model	CPT	QGT
Success rates	92.53%	97.70%

When decision maker T executes the classic Markov dynamic decision, all the current situations are taken into consideration. For example, to calculate the probability of decision maker **T** taking strategy **p** is to add the elements of the first and third lines of $\psi \left(t\right)$, and the same applies to other cases, namely:

17 $${p}_{p}={\psi }_{pp}+{\psi }_{yp}$$

#### Cumulative prospects theoretical (CPT) model

Let $\left\{a\right\}=\{{a}_{1},{a}_{2}\dots {,a}_{n}\}$ is a set of n possible actions,for each action ${a}_{i}$, the possible state set is defined as $\left\{{x}_{i}\right\}=\{{x}_{i,1},{x}_{i,2}\dots {,x}_{i,m}\}$, where ${x}_{i,j}\in R$, and $i=1,\dots ,n,j=1,\dots ,m.$ The probability of each state is expressed as ${p}_{i,j}=p({x}_{i,j}).$ and satisfies that $\sum_{j}^{m}{p}_{i,j}=1$, definition $u({x}_{i,j},{a}_{i})$ is the utility function of each pair of actions-state, then under each decision ${a}_{i}$, the possible prospect can be expressed as ${P}_{i}=(u\left({a}_{i}\right),{p}_{i})$, where $u\left({a}_{i}\right)={[u\left({x}_{i,1},{a}_{i}\right),u\left({x}_{i,2},{a}_{i}\right),\dots ,u\left({x}_{i,m},{a}_{i}\right)]}^{T}$ is the utility vector defined on the possible state set,${p}_{i}={[p\left({x}_{i,1}\right),p\left({x}_{i,2}\right),\dots ,p\left({x}_{i,m}\right)]}^{T}$ is the probability vector corresponding to $\left\{{x}_{i}\right\}$, and the expected utility $U$ of each decision can be written as $U\left({a}_{i}\right)=U\left({P}_{i}\right)=\sum_{j=1}^{m}u\left({x}_{i,j},{a}_{i}\right)p\left({x}_{i,j}\right)$.

**Figure 5:**
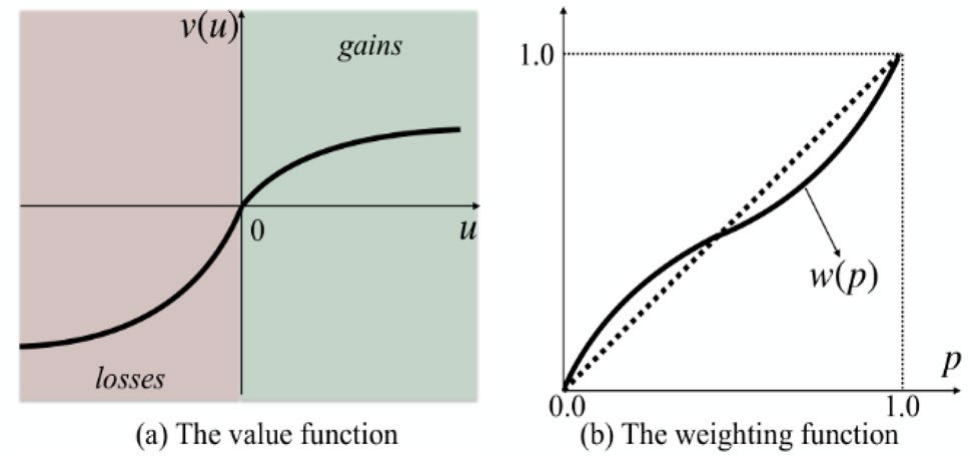
Examples of the value function and weighting function.

Cumulative Prospect Theory (CPT), proposed by Kahneman and Tversky, expounds many biased or irrational human behaviors in a unified way. Compared with the traditional Expected Utility Theory (EUT), CPT introduces two additional concepts in the definition of prospect: (1) P: value function $V$ defined in utility, (2) decision weight function $\pi$ defined in cumulative probability (as shown in Fig. 5). Each action is evaluated by the following equation:

18 $$V\left( {a_{i} } \right) = V\left( {P_{i} } \right) = \sum\limits_{j = 1}^{m} v \left( {u^{ + } \left( {x_{i,j} ,a_{i} } \right)} \right)\pi_{j}^{ + } + v\left( {u^{ - } \left( {x_{i,j} ,a_{i} } \right)} \right)\pi_{j}^{ - }$$

where, the function $V$ is a strictly increasing function, and ${u}^{+}$ and ${u}^{-}$ are the gains and losses of $u$ compared with the reference utility ${u}_{0}$. Decision weight is defined as:

19 $$\begin{aligned} & \pi_{m}^{ + } = w^{ + } \left( {p\left( {x_{i,m} } \right)} \right),\quad \pi_{m} = w^{ - } \left( {p\left( {x_{i,m} } \right)} \right),\;\pi_{j}^{ + } = w^{ + } \left( {\sum\limits_{k = j}^{m} p \left( {x_{i,k} } \right)} \right) - w^{ + } \left( {\sum\limits_{k = j + 1}^{m} p \left( {x_{i,k} } \right)} \right), \hfill \\ & \pi_{j}^{ - } = w^{ - } \left( {\sum\limits_{k = j}^{m} p \left( {x_{i,k} } \right)} \right) - w^{ - } \left( {\sum\limits_{k = j + 1}^{m} p \left( {x_{i,k} } \right)} \right), \hfill \\ & \forall j = 1, \ldots ,m - 1 \, \hfill \\ \end{aligned}$$

where, ${w}^{\pm }$ is a strictly increasing function, in general, $V(u)$ is convex for $u\ge {u}_{0}$ (gain), when $u\le {u}_{0}$ (loss), $V(u)$ is concave and the loss is steeper than the gain. Figure 5a shows an example of a value function when ${u}_{0}=0$ is set as a reference utility. Many experimental studies have shown that the representative function form of $V$ and $w$ can be written

$$v(u) \, = \left\{ {\begin{array}{*{20}l} {\left( {u - u_{0} } \right)^{\alpha } ,} \hfill & {{\text{ if }}u \ge u_{0} } \hfill \\ { - \lambda \left( {u_{0} - u} \right)^{\beta } ,} \hfill & {{\text{ if }}u < u_{0} } \hfill \\ \end{array} } \right.$$

20 $$w^{ + } (p) \, = \frac{{p^{\gamma } }}{{\left( {p^{\gamma } + (1 - p)^{\gamma } } \right)^{1/\gamma } }},\;\;w^{ - } (p) \, = \frac{{p^{\delta } }}{{\left( {p^{\delta } + (1 - p)^{\delta } } \right)^{1/\delta } }}.$$

where, $\alpha ,\beta ,\gamma ,\delta \in (\mathrm{0,1}]$, and $\lambda \ge 1$, in Fig. 5b, this decision weight function can well describe the observed behavior that humans tend to overestimate the occurrence of low probability events and underestimate the occurrence of high probability events.

CPT model assumes that the decision-maker chooses the behavior that produces the maximum value defined in (18), that is,

21 $${a}^{*}={argmax}_{{a}_{i}\in \{a\}}\left\{V\left({a}_{i}\right)\right\}=\left\{V\left({a}_{1}\right),\dots ,V\left({a}_{n}\right)\right\}$$

#### Quantum game theoretical (QGT) model

In the QGT model, decision maker T is in a superposition state before observing I, after observation, the superposition state is transformed into a possible ground state, the probability of its transformation is the square of the magnitude of the probability amplitude (${\psi }_{ij}$) and it satisfies the uniformity, as shown in Eq. (22) :

22 $$\sum\limits_{{\text{i}}} {\sum\limits_{{\text{j}}} {\left\| {\psi_{ij} } \right\|^{2} = 1} }$$

In the QGT model, the initial behavior state vector of the decision maker is

23 $${\psi }_{0}\left(0\right)=\left[\begin{array}{c}\begin{array}{c}\begin{array}{c}0.5\\ 0.5\end{array}\\ 0.5\end{array}\\ 0.5\end{array}\right] {,\psi }_{1}\left(0\right)=\left[\begin{array}{c}\begin{array}{c}\begin{array}{c}\frac{1}{\sqrt{2}}\\ \frac{1}{\sqrt{2}}\end{array}\\ 0\end{array}\\ 0\end{array}\right],{\psi }_{2}\left(0\right)=\left[\begin{array}{c}\begin{array}{c}\begin{array}{c}0\\ 0\end{array}\\ \frac{1}{\sqrt{2}}\end{array}\\ \frac{1}{\sqrt{2}}\end{array}\right]$$

In the same way as the classical Markov dynamic model, after time t, the initial vector will evolve into the final vectors ${\psi }_{0}\left(t\right),{\psi }_{1}\left(t\right),{\psi }_{2}\left(t\right)$, which also represent the completion of the decision. This dynamic process can be described by the solution of Schrodinger equation:

24 $$\psi \left(t\right)={e}^{-it{H}_{A}}\psi \left(0\right)$$

where, ${H}_{A}$ is a Hamiltonian matrix, which is the key to the solution of the equation,It is similar to the construction of the strength matrix, and finally the Hamiltonian matrix is described as:

25 $${H}_{A}=\left[\begin{array}{cc}{H}_{Ap}& 0\\ 0& {H}_{Ay}\end{array}\right], \;\;{H}_{Ai}=\left[\begin{array}{cc}{u}_{i}& 1\\ 1& {-u}_{i}\end{array}\right]$$

Different from the classical Markov decision model, utility functions ${u}_{i}$ range from $-1$ to 1 . At the same time, in the decision model of QGT, we should consider the influence of irrational behavior, which Busemeyer called "cognitive disorder"^34^. The relationship between belief and behavior can be expressed by the following matrix:

26 $$H_{B}=-\frac{\gamma }{{\sqrt 2 }}\left[ {\begin{array}{*{20}c} 1 & 0 & 1 & 0 \\ 0 & {-1} & 0 & 1 \\ 1 & 0 & {-1} & 0 \\ 0 & 1 & 0 & 1 \\ \end{array} } \right]$$

The irrational behavior matrix is added to the decision model of QGT, and a new Hamiltonian matrix ${(H}_{A}+{H}_{B})$ is constructed and brought into the solution of Schrodinger equation.

However, the above-mentioned quantum game model method has some problems: when decision maker T knows the intent of I, it can find the solutions of ${\psi }_{1}\left(t\right)$ and ${\psi }_{2}\left(t\right)$, When I's intention is unknown, the solution of ${\psi }_{0}\left(t\right)$ cannot be obtained by the above method, the reasons are as follows: (1) The interaction is not fully considered, and decision-maker T only considers it from its own profit dimension, thus ignoring it. In fact, in the case of uncertainty about I's intention, I's benefits should be considered. (2) The utility function is related to the profit value of both parties, so the profit of I need not be considered when determining the intention of I, but it needs to be considered when the intention of I is uncertain, in the past, the utility function obtained ${u}_{p}={u}_{y}=u(5)$ according to Eq. (16), but in fact, ${u}_{p}$ is not necessarily equal to ${u}_{y}$, so the utility function needs to be processed in the next improvement process.

When the decision maker T doesn't know I's intention, the QGT model (Eqs. 24 and 25) built before is improved, and the benefits of decision maker T and I are considered, make the following improvements: reconstruct the Hamiltonian matrix, and add the benefits of I when constructing, and get:

$${H}_{00}={H}_{01}+{H}_{02};\;\;\;{H}_{01}=\left[\begin{array}{cc}{H}_{p}& 0\\ 0& {H}_{y}\end{array}\right],$$

$${H}_{p}=\frac{1}{\sqrt{1+{{u}_{p}}^{2}}}\left[\begin{array}{cc}{u}_{p}& 1\\ 1& {-u}_{p}\end{array}\right],\;\;\;{H}_{y}=\frac{1}{\sqrt{1+{{u}_{y}}^{2}}}\left[\begin{array}{cc}{u}_{y}& 1\\ 1& {-u}_{y}\end{array}\right]$$

27 $${H}_{02}=\left[\begin{array}{cc}{H}_{p1}& 0\\ 0& {H}_{y1}\end{array}\right],\;\;{H}_{p1}=\frac{1}{\sqrt{1+{{u}_{p1}}^{2}}}\left[\begin{array}{cc}{u}_{p1}& 1\\ 1& {-u}_{p1}\end{array}\right],\;\;\;{H}_{y1}=\frac{1}{\sqrt{1+{{u}_{y1}}^{2}}}\left[\begin{array}{cc}{u}_{y1}& 1\\ 1& {-u}_{y1}\end{array}\right]$$

where, ${H}_{00}$ is a newly constructed Hamiltonian matrix; ${H}_{01}$ is the profit of decision maker T; ${H}_{02}$ is the profit of I; ${H}_{p1}$/${H}_{y1}$ is Hamiltonian matrix when I adopts strategy **p**/**y**; ${u}_{p1}$/${u}_{y1}$ is when I takes strategy **p**/**y**, the difference between the profit of I earned by decision maker T adopting strategy **p** and the profit of I earned by decision maker T adopting strategy **y**.

The newly constructed matrix above solves the first problem, and then, it needs to solve the problem of oversimplification of utility function, that is, how to define a utility function that can effectively reflect the difference between the benefits of decision makers. We choose to use the value function in expectation theory, such as Eq. (28), and make proper normalization to meet the requirements of QGT model for the value range of utility function.

28 $$\begin{aligned} {u}_{p} &=\frac{2}{1+{e}^{{-D}_{p}}}-1;\;\;{u}_{y}=\frac{2}{1+{e}^{{-D}_{y}}}-1, \\ {u}_{p1}& =\frac{2}{1+{e}^{{-D}_{p1}}}-1; \;\;{u}_{y1}=\frac{2}{1+{e}^{{-D}_{y1}}}-1.\end{aligned}$$

where, ${D}_{p}$ represents when I adopts strategy **p**, the difference in value between the decision maker T who chooses to take strategy **p**, profit is 10, and who chooses to take strategy **y**, profit is 5, ${D}_{p}$ is normalized with a hyperbolic tangent function similar to logistic regression: ${D}_{p}$ = ${10}^{a}-{5}^{a}$; ${D}_{y}$ is when I adopts strategy **y**, the difference in value between the decision maker T who chooses to take strategy **p**, profit is 25, and who chooses to take strategy **y**, profit is 20, in a similar way, ${D}_{y}$ = ${25}^{a}-{20}^{a}$, power $a$ is the risk aversion index of decision maker T in its own income dimension, with the value between 0 and 1. ${D}_{p1}$ is when I adopts strategy **p**, the difference in value between the decision maker T who chooses to take strategy **p**, I gets the profit is 10, and who chooses to take strategy **y**, I gets the profit is 25: ${D}_{p1}$ = ${10}^{b}-{25}^{b}$; ${D}_{y1}$ is when I adopts strategy **y**, the difference in value between the decision maker T who chooses to take strategy **p**, I gets the profit is 5, and who chooses to take strategy **y**, B gets the profit is 20, ${D}_{y1}$ = ${5}^{b}-{20}^{b}$, power $b$ is the risk aversion index of decision maker T in the income dimension of I. In the game in real time, in order to ensure the benefits of decision maker T, the benefits of decision maker T are greater than those of decision maker I,so the choice between $a$ and $b$ is $0<b<a<1$. In this paper, $b= a/4$, because the separation effect is most obvious at this time.

## Case study

**Figure 6:**
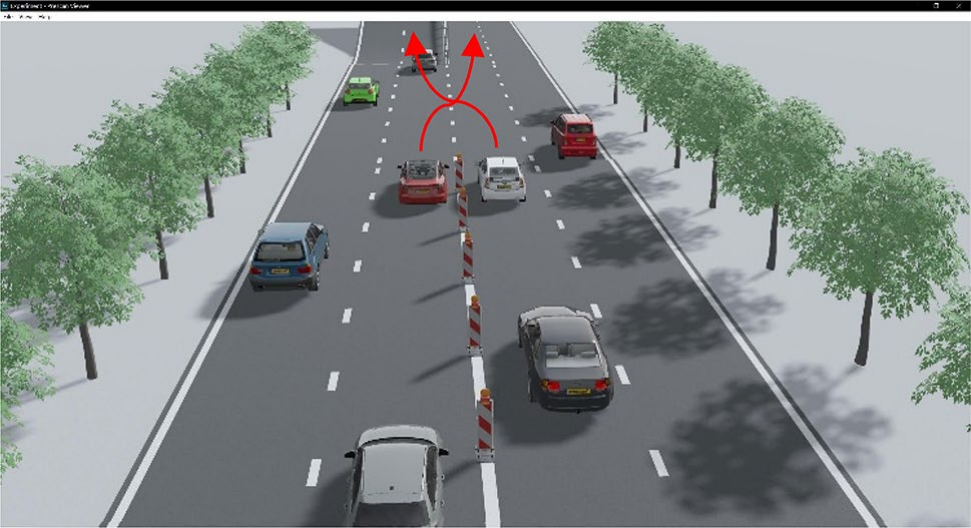
Game scene in complex traffic environment.

In this paper, two car game scene as shown in Fig. 6, white car for target vehicle, red car for interact vehicle. The two cars were driving side by side at the intersection of the viaduct and the side road, target vehicle need to turn left to the viaduct, interact vehicle need to turn right to the side road, the game scenario is created. From the first perspective of target vehicle, there are three situations: seeing the interact vehicle trying to speed up and pass, seeing the interact vehicle slowing down and yield, and not being sure of the interact vehicle’s intent. What kind of decision does the target vehicle make in three situations?

According to the classic Markov dynamic decision model (Eq. 14), when the opponent's vehicle intention is uncertain, the probability of the target vehicle adopting accelerated overtaking is the same as the probability of the target vehicle when the opponent wants to accelerate or when the opponent wants to decelerate, which cannot explain the separation effect and obviously does not conform to the actual situation, so the process is omitted.

### Simulation analysis based on CPT model

There are two actions for defining vehicles: pass and yield, that is, $\left\{a\right\}=\{{a}_{p},{a}_{y}\}$,under the ${a}_{p}$ decision, the target vehicle needs to test the possibility that the interact vehicle will not yield, which can force the target vehicle to brake without passing by law. However, for ${a}_{y}$ decision, we can assume that it is always successful. Therefore, the prospects of ${a}_{p}$ and ${a}_{y}$ are:

29 $$\begin{array}{*{20}l} {P_{{a_{p} }} = \left\{ {\left( {u\left( {\hat{\xi }_{I,y} ,\hat{\xi }_{T,p} } \right),p_{I,y} } \right),\left( {u\left( {\hat{\xi }_{1,ny} ,\hat{\xi }_{T,p} } \right),1 - p_{I,y} } \right)} \right\}} \hfill \\ {P_{{a_{y} }} = \left\{ {\left( {u\left( {\hat{\xi }_{I,ny} ,\hat{\xi }_{T,y} } \right),1.0} \right)} \right\}} \hfill \\ \end{array}$$

where, $\{{\xi }_{I}^{t},{\xi }_{T}^{t}\}$ is the historical track set of vehicles, I indicates the interact vehicle, T indicates the target vehicle, ${p}_{I,y}$ indicates the yield probability of interact vehicle, ${\widehat{\xi }}_{I,y}$ and ${\widehat{\xi }}_{I,ny}$ is divided into yield track and non-yield track of interact vehicle. ${\widehat{\xi }}_{T,p}$ and ${\widehat{\xi }}_{T,y}$ divide into the pass track and yield track of target vehicle. Make ${u}_{0}=0$. Looking back at the CPT models defined in (18)–(21), the CPT values of target vehicle under different decisions can be written as:

30 $$\begin{aligned} V\left( {a_{p} } \right) & = v\left( {u^{ + } \left( {\hat{\xi }_{1,y} ,\hat{\xi }_{T,p} } \right)} \right)\left( {w^{ + } (1.0) - w^{ + } \left( {p_{I,y} } \right)} \right) + v\left( {u^{ + } \left( {\hat{\xi }_{1,ny} ,\hat{\xi }_{T,p} } \right)} \right)w^{ + } \left( {p_{I,y} } \right) \hfill \\ & = \left( {u\left( {\hat{\xi }_{1,y} ,\hat{\xi }_{T,p} } \right)} \right)^{\alpha } \left( {1 - \frac{{p_{I,y}^{\gamma } }}{{\left( {p_{I,y}^{\gamma } + \left( {1 - p_{I,y} } \right)^{\gamma } } \right)^{1/\gamma } }}} \right) + \left( {u\left( {\hat{\xi }_{I,ny} ,\hat{\xi }_{T,p} } \right)} \right)^{\alpha } \frac{{p_{I,y}^{\gamma } }}{{\left( {p_{I,y}^{\gamma } + \left( {1 - p_{I,y} } \right)^{\gamma } } \right)^{1/\gamma } }}, \hfill \\ \end{aligned}$$

$$V\left( {a{}_{y}} \right) = v\left( {u^{ + } \left( {\hat{\xi }_{1,ny} ,\hat{\xi }_{T,y} } \right)} \right)w^{ + } \left( {1.0} \right) = \left( {u\left( {\hat{\xi }_{1,ny} ,\hat{\xi }_{T,y} } \right)} \right)^{a} .$$

Then write the decision of target vehicle as

31 $$a^{*} = \arg {\max}_{{a \in \left\{ {a_{p} ,a_{y} } \right\}}} \left\{ {V\left( {a_{p} } \right),V\left( {a_{y} } \right)} \right\}$$

**Figure 7:**
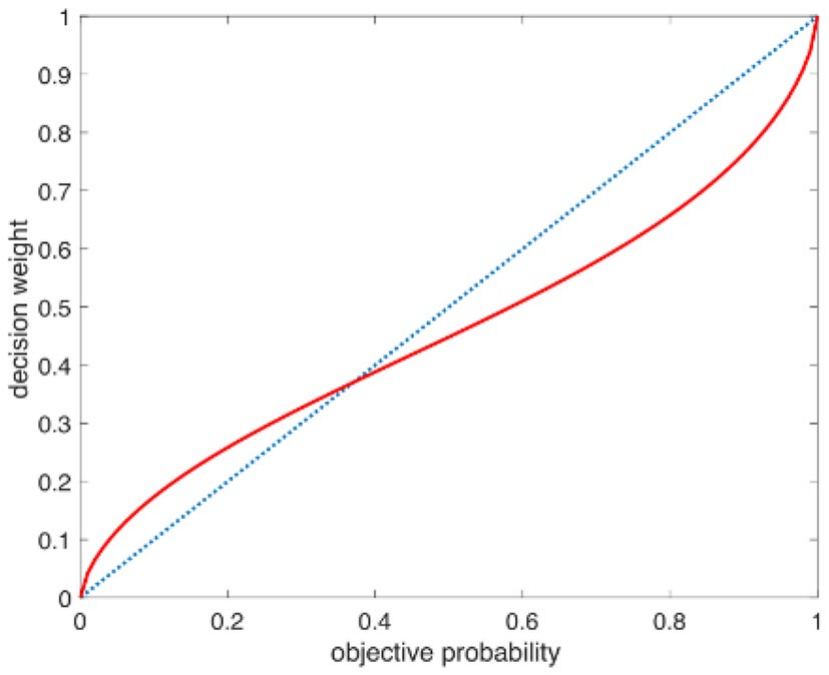
The learned decision weighting function (red curve).

The method in reference^45^ obtains the parameters in CPT by Inverse Reinforcement Learning (IRL), assuming that $u$ is a linear combination of some characteristics, including speed, acceleration, emergency braking and safety. The learned decision weighting function is shown in Fig. 7. CPT model does capture people's choice pattern, that is, low probability events are often overestimated, while high probability events are often underestimated. This result is consistent with the research on human behavior in other fields such as economics, investment and waiting paradox.

From the simulation results (Fig. 7), the CPT model can solve the irrational problem in automatic driving decision-making, but this paper would like to put forward three different views: (1) The probability value calculated by the CPT is based on the classical probability, and does not take into account the situation when the superposition state is generated,for example, when setting the action set, there are only two actions: pass and yeild, but in actual scenes, many interactive vehicles will be hesitant. (2) When the cumulative prospect theory uses IRL to learn parameters, it is assumed that the interact vehicle will not yield when the target vehicle wants to pass, and the original initial speed of the interact vehicle will remain unchanged, this assumption is not in line with the actual situation,if the interact vehicle does not yield, the original speed should be increased to prompt the target vehicle, so the result obtained at this time will be different from the actual situation. (3) CPT assumes that when the interact vehicle yields, the target vehicle will pass 100%, this assumption is completely rational and does not conform to the actual situation.

### Simulation analysis based on QGT model

**Figure 8:**
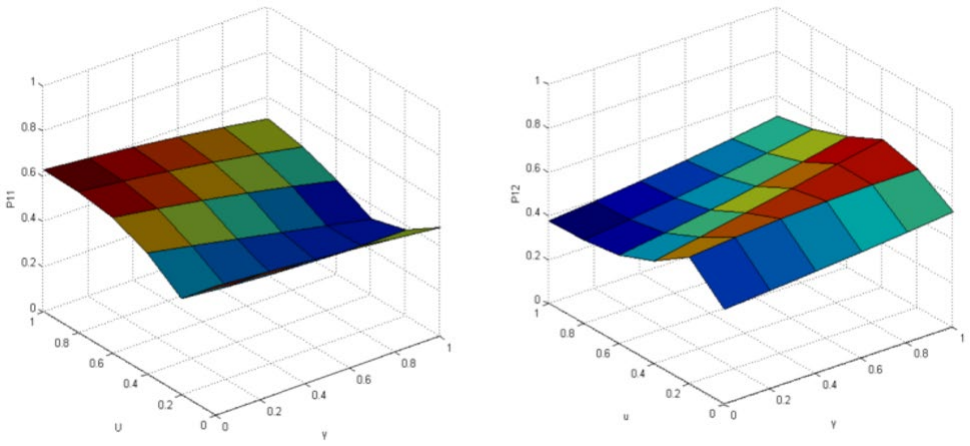
Probability of the target vehicle pass (left:P11)/yeild (right:P12) when knowing that interact vehicle intends to pass (t = π/2).

**Figure 9:**
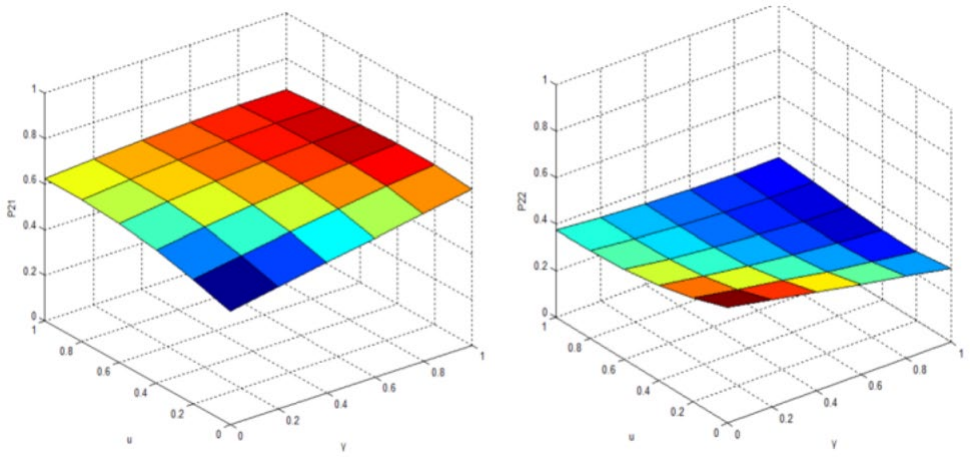
Probability of the target vehicle pass (left:P21)/yeild (right:P22) when knowing that interact vehicle intends to yeild (t = π/2).

Compared with the classical Markov decision model, the results obtained by the QGT model can better explain the separation effect. MATLAB simulation platform is used for simulation, when the interact vehicle’s intention is clear, the standard quantum game model is used. The variable parameters are quantum entanglement factor γ and utility function $u$. The results are shown in Figs. 8 and 9.

**Figure 10:**
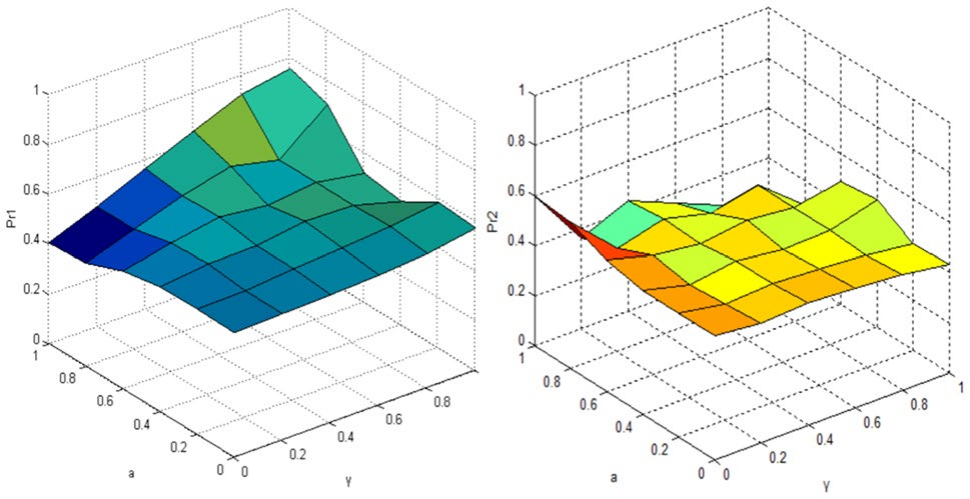
Probability of the target vehicle pass (left:Pr1)/yeild (right:Pr2) when the intention of interact vehicle is uncertain after optimization (t = π/2).

When the intention of the interact vehicle 's vehicle is uncertain, the probability of pass/yeild the vehicle of the target vehicle is simulated by improved QGT, and the benefits of both parties are considered from the perspective of the opponent's vehicle, the parameter variables are quantum entanglement factor γ and risk aversion index $a$, and the results are shown in Fig. 10.

In the improved model, when quantum entanglement is 0, the probability of pass or yeild the target vehicle collapses to the classical model, which is 0.5, and then fluctuates with the increase of quantum entanglement and benefit function, in the case of maximum quantum entanglement, the improved QGT model is more inclined to pass (probability value is 0.75), the irrational behavior and the interaction between the two vehicles are considered in the QGT model, which is more suitable for the actual situation than the CPT model.

## Experimental analysis

**Figure 11:**
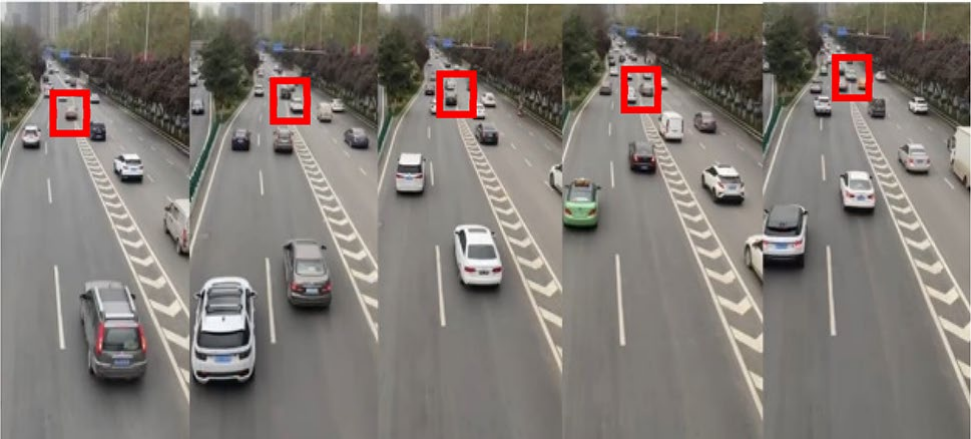
In the interaction scenes collected from the real road data set, the area where the game takes place is marked with the red box.

In order to verify the effectiveness of CPT and QGT, the experimental data set scene is similar to the simulation model, in the real scene, vehicles on the two main roads are allowed to change lanes in the red box, so this is the center of game decision-making. We trained and tested two models on a data set (Fig. 11) containing 348 pairs of interacting trajectories with a sampling frequency of 10 Hz. To learn more generalized results, we slice the trajectories into frames with a fixed length using moving windows. Each frame contains the trajectories in 1 s. Thus, all 348 pairs of interacting trajectories generate 13,920 frames.

The verification results of the two models are shown in the following table (Table 2). The results show that the decision-making accuracy of the QGT model is higher than CPT model. The main reasons are as follows: (1) The probability value calculated by CPT model is based on the classical probability calculation, without considering the situation of superposition state. For example, when setting the action set, there are only two actions of speeding through and slowing down, but there is no state between them, therefore, this method does not fundamentally solve the irrational decision-making problem; (2) In reference^45^, inverse reinforcement learning (IRL) for parameters in CPT did not take into account the influence of interaction between the two sides of the interaction, resulting in a relatively low success rate; (3) Compared with CPT, QGT takes into account the superposition state in the action set, which is more consistent with the actual situation without completely rational assumption.

However, we also need to demonstrate the benefits of QGT in terms of data efficiency. Neural network model achieves good results in specific scenarios through a large number of data-driven methods^45^, while QGT does not require a large number of data-driven methods. If our results are similar to or even more advantageous than the neural network model, the QGT proposed by us will have more theoretical value.

The neural network algorithm is applied to the data set (Fig. 11). To achieve better performance for the learning-based model, we have conducted two sets of experiments for the training of the neural network:

- Experiment 1: randomly shuffle all the trajectory pairs and select 80% of them for training and the other 20% for testing. The success rate is 65% for testing.
- Experiment 2: directly shuffle all frames for the neural network and randomly select 80% for training and 20% for testing. The success rate is 97% for testing.

The large discrepancy between the testing accuracies of the two experiments with the NN model is mainly due to the over-fitting problem cause by the data insufficiency. In experiment 1, it showed that the NN model learned on 80% of the trajectory pairs cannot be well generalized to other interaction pairs.

Table 3 shows the results of the comparison. The results show that: QGT model compared to neural network model, the result is close to and the QGT model does not need to be driven by a large amount of data, which makes the QGT model more efficient than the neural network model.

**Table 3:** Comparison of the success rates in NN model and QGT model.

Model	NN	QGT
Success rates	97.02%	97.70%

Success rates: the percentage of correct predictions among all test examples.

## Conclusion and prospect

In this study, the QGT model was used to analyze the interaction between two vehicles, compared with the classical Markov dynamic decision model. This model is more practical and successfully explains the separation effect. Compared with the CPT model, the QGT model takes the superposition state in the action set into consideration and abandons the assumption of complete rationality, which is more consistent with the actual situation. When the opponent's vehicle intention is known, only the uncertainty and irrational behavior in the environment are considered, and the probability value fitting the actual situation is obtained. Otherwise, the profit value of both sides of the game will be also considered from the perspective of the opponent's vehicle, and the actual result is obtained. According to the case analysis, the QGT model owns more advantages than the classical Markov dynamic decision model and CPT model in explaining the uncertainty and irrational behavior and interaction of other traffic participants. The CPT model and QGT model are further verified by data sets, showing that the QGT model has more advantages in dealing with game scene decisions. At the same time, compared to neural network model, the result of QGT model is close to and the QGT model does not need to be driven by a large amount of data, which makes the QGT model more efficient than the neural network model.

In the following work, more complex traffic scenes (such as real road data sets) are cited, and their interaction with other traffic participants in automatic driving is further explored by combining quantum theory with deep learning.

Although the application research is only carried out at the simple two-car game scenario, the research method adopted is also instructive and referential for more complex scenes in automatic driving. This paper firstly attempt to apply QGT to automatic driving, providing a new reference frame for the study of decision-making problem of bounded rational behavior interaction of human traffic participants. We believe that with the further development of quantum decision theory and the continuous exploration of researchers, its application in autonomous driving will be more popular and in-depth.
